# Supplementary material for: CLUH granules coordinate translation of mitochondrial proteins with mTORC1 signaling and mitophagy
Source: EMBO J. 2020 Mar 9;39(9):e102731. doi: 10.15252/embj.2019102731 (PMC7196838; doi:10.15252/embj.2019102731)
Supplement: Supplementary file 1 — Appendix [file EMBJ-39-e102731-s001.pdf]

TABLE OF CONTENT

Figure Appendix S1. Ribopuromycylation control experiments**.**

Figure Appendix S2. Whole cell images of experiments shown in Figure 3.

Figure Appendix S3. CLUH association with target mRNAs is lost upon HHT treatment.

Figure Appendix S4. 1D pathway enrichments of transcriptomic and proteomic profiles in primary hepatocytes.

Figure Appendix S5. Mitochondrial integrated stress response is not activated in absence of CLUH.

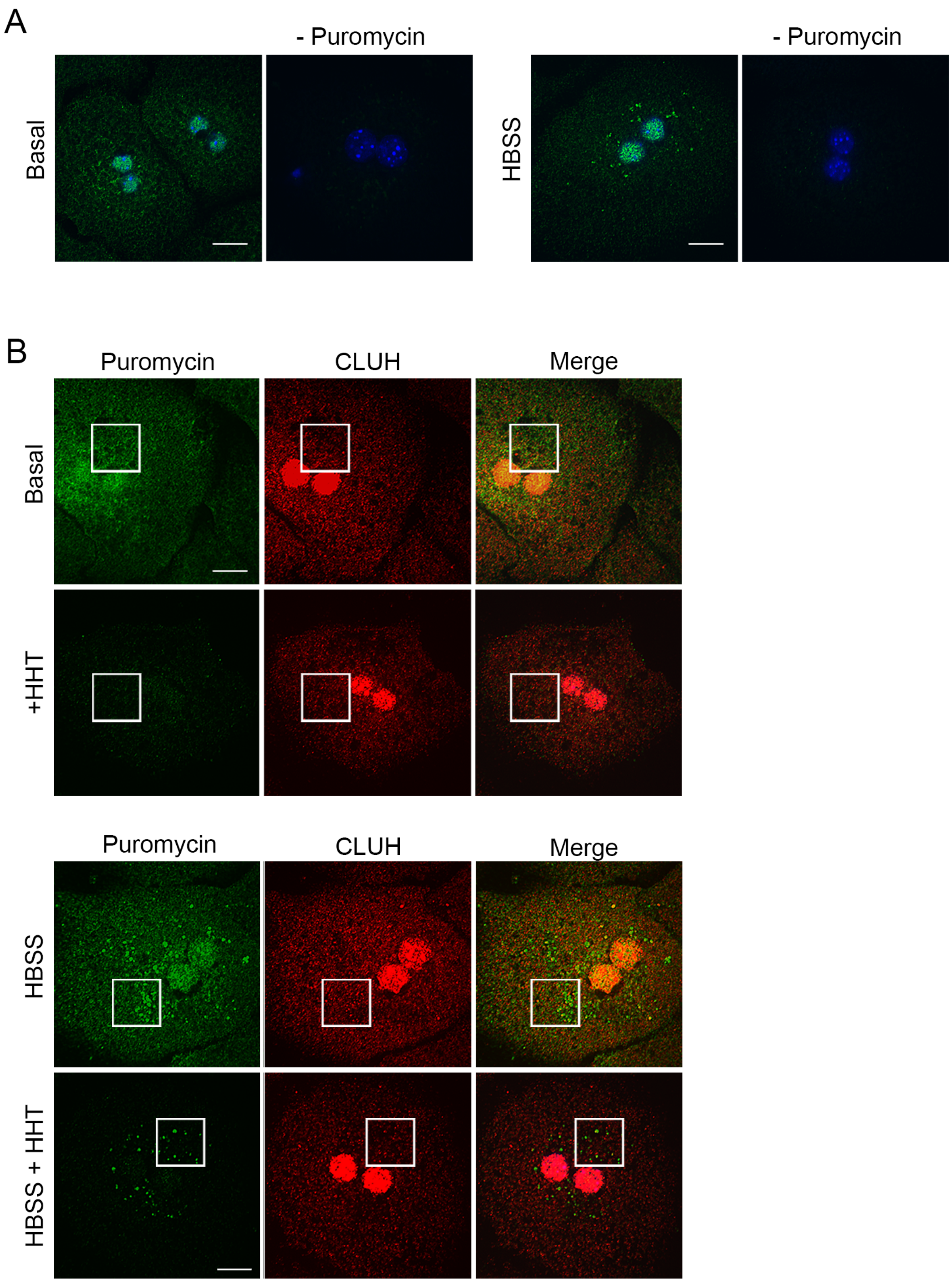

Figure Appendix S1. Ribopuromycylation control experiments.

A Confocal images of primary hepatocytes after ribopuromycylation treated with or without puromycin and stained with anti-puromycin antibody. Scale bar, 10 µm.

B. Confocal images of primary hepatocytes after ribopuromycylation cultured in indicated conditions with or without HHT and stained with indicated antibodies. Boxes in these images correspond to the enlargement shown in Figure 2C. Scale bar, 10 µm.

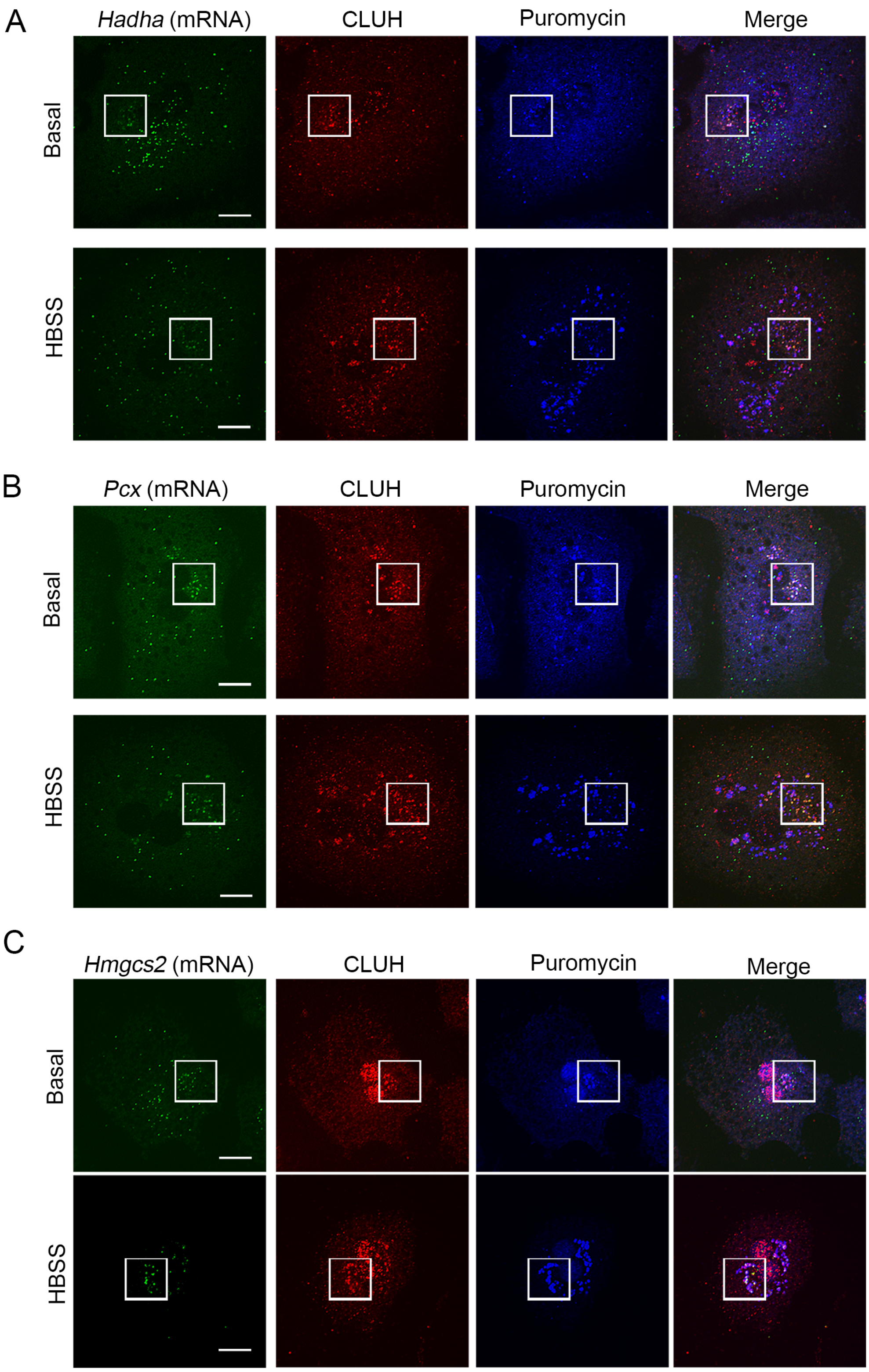

Figure Appendix S2. Whole cell images of experiments shown in Figure 3.

A-C Confocal images of primary hepatocytes after ribopuromycylation combined with mRNA *in situ* hybridization for (A) *Hadha*, (B) *Pcx* and (C) *Hmgcs2* and stained with the indicated antibodies. Boxes in these images correspond to enlargement shown in Figure 3. Scale bar, 10 µm.

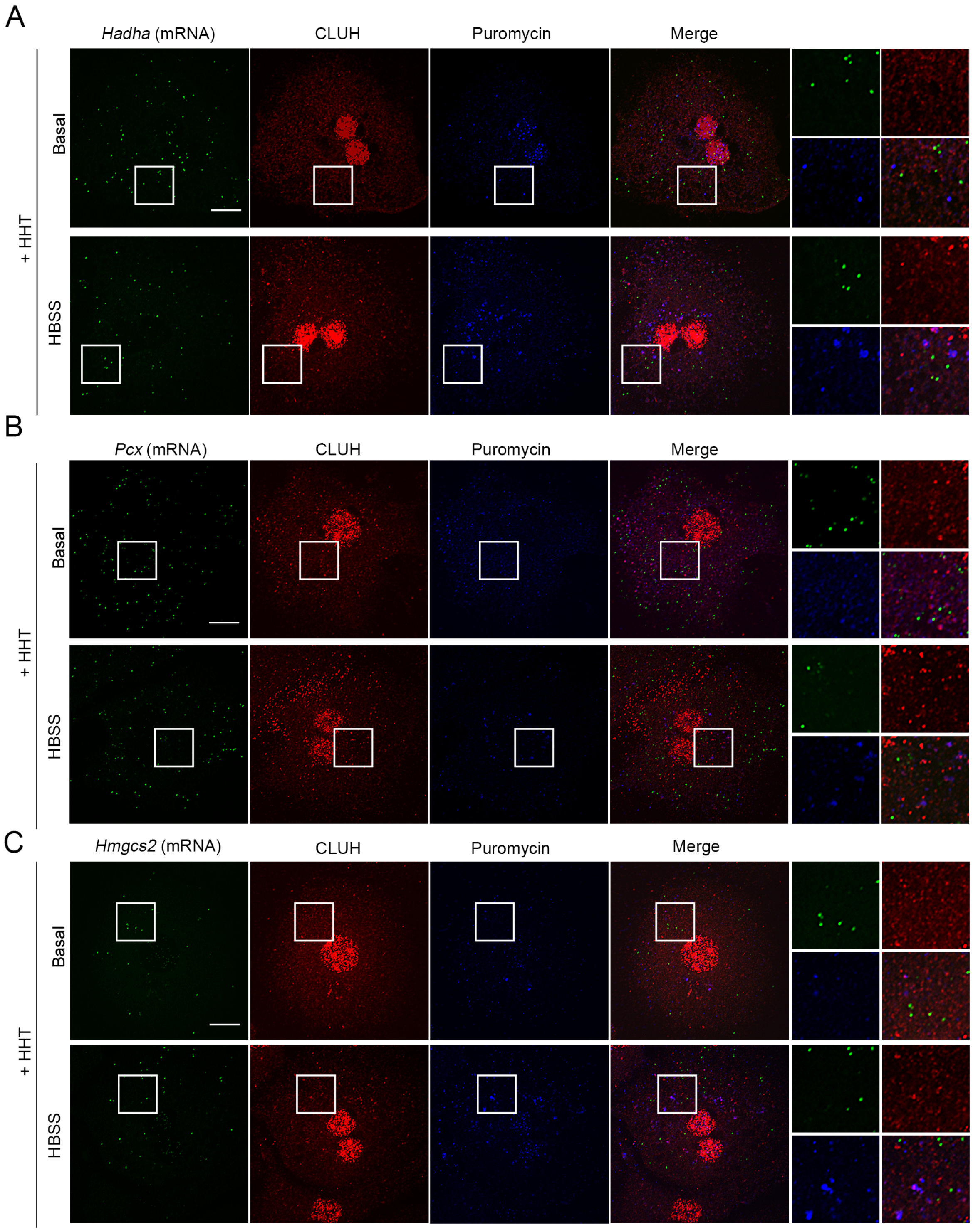

Figure Appendix S3. CLUH association with target mRNAs is lost upon HHT treatment. A-C Confocal images of primary hepatocytes cultured in the indicated media incubated with HHT before ribopuromycylation, stained with the indicated antibodies combined with mRNA *in situ* hybridization for (A) *Hadha*, (B) *Pcx* and (C) *Hmgcs2*. Boxes in these images are shown 2.5x magnified on the right side in all channels and merge. Scale bar, 10 μm.

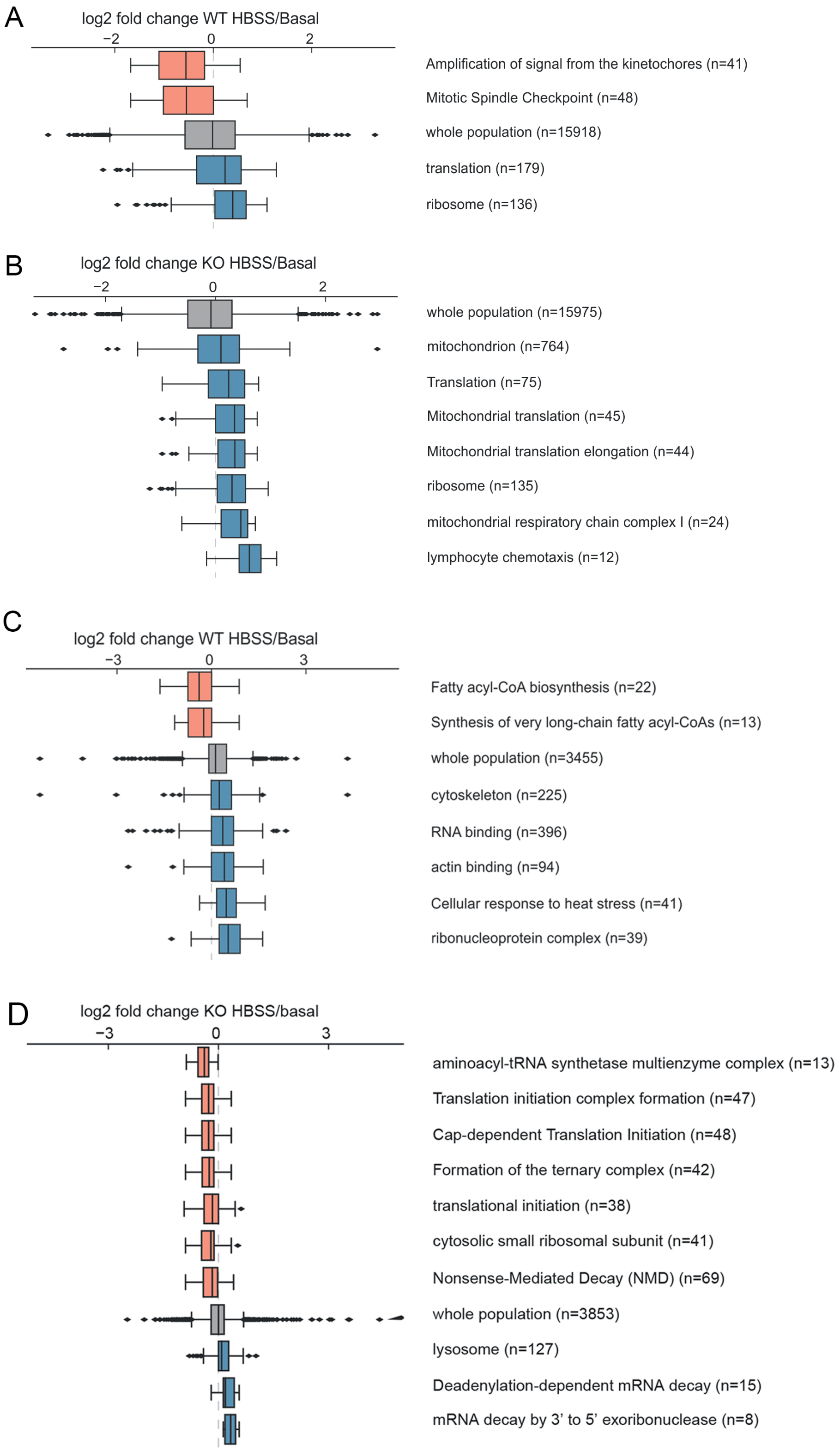

Figure Appendix S4. 1D pathway enrichments of transcriptomic and proteomic profiles in primary hepatocytes.

A-D Box plots showing the distribution of fold changes of enriched pathways selectively found in transcriptomics (A, B) and proteomics (C, D) analysis of WT and *Cluh* KO hepatocytes based on 1D enrichment analysis (FDR < 0.02).

A

Gene	Fold change KO/WT Basal	P-value Basal	Fold change KO/WT HBSS	P-value HBSS
Atf4	-1.1	0.458042	-1.31	0.109528
Atf5	-1.2	0.292927	-1.27	0.365616
Psat1	1.23	0.392629	1.35	0.250093
Phgdh	1.14	0.633075	1.19	0.61792
Mthfd2	-1.25	0.298401	-1.2	0.451419

B

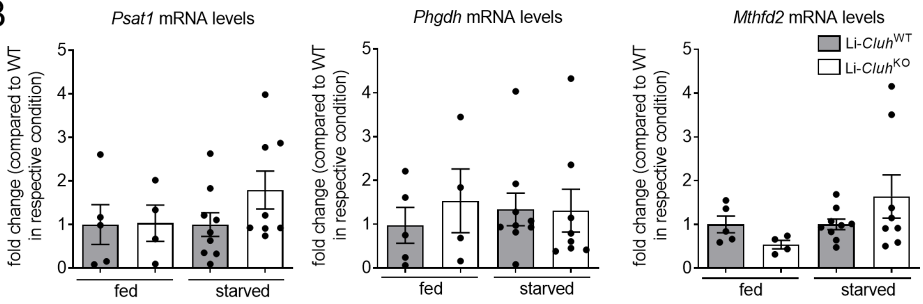

Figure Appendix S5. Mitochondrial integrated stress response is not activated in absence of CLUH.

A Fold change and respective p-value of indicated genes extracted from transcriptomic analysis.

B mRNA levels of indicated genes in livers from fed and starved Li-*Cluh*^{WT} and Li-*Cluh*^{KO} mice. WT fed: n=5; KO fed: n=4; WT starved: n=9; KO starved: n=8.
