# Supplementary material for: CLUH granules coordinate translation of mitochondrial proteins with mTORC1 signaling and mitophagy
Source: EMBO J. 2020 Mar 9;39(9):e102731. doi: 10.15252/embj.2019102731 (PMC7196838; doi:10.15252/embj.2019102731)
Supplement: Supplementary file 2 — Expanded View Figures PDF [file EMBJ-39-e102731-s002.pdf]

Expanded View Figures

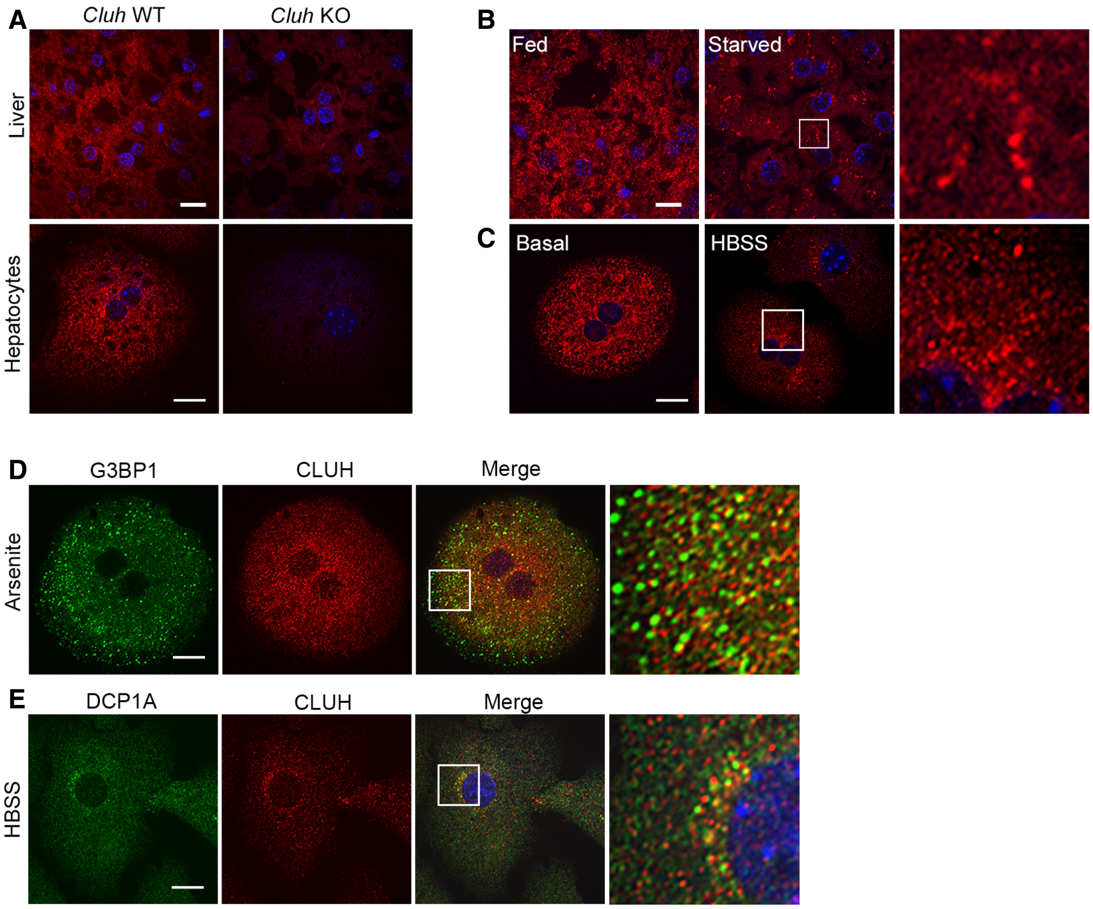

Figure EV1. CLUH forms granules upon starvation.

- A Confocal images of liver cryosections and primary hepatocytes of Li-*Cluh*^{WT} and Li-*Cluh*^{KO} mice stained with anti-CLUH antibody. Scale bar, 10 μm.
- B Confocal images of liver cryosections of fed and starved Li-*Cluh*^{WT} and Li-*Cluh*^{KO} mice stained with anti-CLUH antibody. Right panels show 6.5× magnified boxed areas. Scale bar, 10 μm.
- C Confocal images of primary hepatocytes cultured in indicated media and stained with anti-CLUH antibody. Right panels show 4.5× magnified boxed areas. Scale bar, 10 μm.
- D, E Confocal images of primary hepatocytes cultured in indicated media and stained with (D) anti-G3BP1 or (E) anti-DCP1A and anti-CLUH antibodies. Right panel shows 5× enlargement of indicated area. Scale bar, 10 μm.

© 2020 The Authors

Figure EV2. Overexpressed CLUH forms CHX resistant granules in HeLa cells.

A Confocal images of WT and *CLUH* KO HeLa cells stained with anti-CLUH antibody. Scale bar, 10 μm.

B Confocal images of HeLa cells downregulated for G3BPs and overexpressing untagged CLUH (marked with asterisks). These images were overexposed to detect cells with CLUH expression at endogenous level CLUH. Asterisks indicate overexpressing cells. Scale bar, 10 μm.

C, D Confocal images of HeLa cells overexpressing untagged CLUH (C) or FLAG-tagged CLUH (D) stained with indicated antibodies. Scale bar, 10 μm.

E Confocal images of HeLa cells overexpressing untagged CLUH treated with or without CHX and stained with the indicated antibodies. Scale bar, 10 μm.

F Quantification of percentage of cells with CLUH granules of experiment shown in (E) (n = 3 independent experiments, > 50 cells per condition per replicate).

G Confocal images of HeLa cells treated with arsenite with or without CHX and stained with anti-G3BP1 antibody. Scale bar, 10 μm.

H Quantification of percentage of cells with G3BP1 granules of experiment shown in (G) (n = 3 independent experiments, > 50 cells per condition per replicate).

I Live imaging of WT and *CLUH* KO HeLa cells transfected with G3BP1-GFP plasmid and treated with arsenite with and without CHX. Cells were recorded for a maximum of 30 min. Scale bar, 10 μm.

J Total number of cells analyzed by live imaging for the indicated experiments. “Positive” indicates a cell which forms G3BP1 granules at the end of the recording.

Data information: In (F, H), data are presented as histograms showing the mean ± SEM. (H) ***$P \leq 0.001$ (Student’s t-test).

© 2020 The Authors

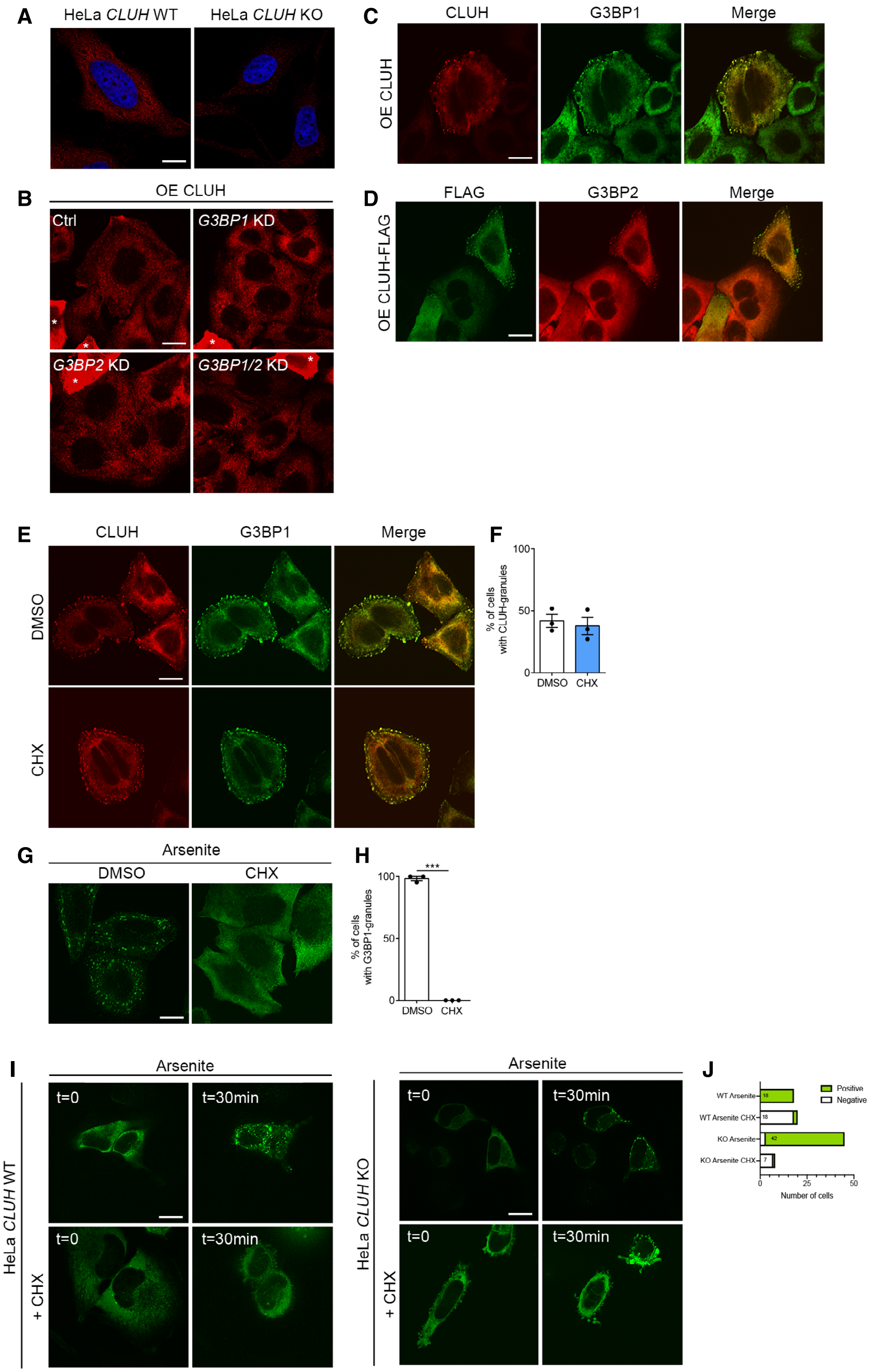

Figure EV2.

 © 2020 The Authors

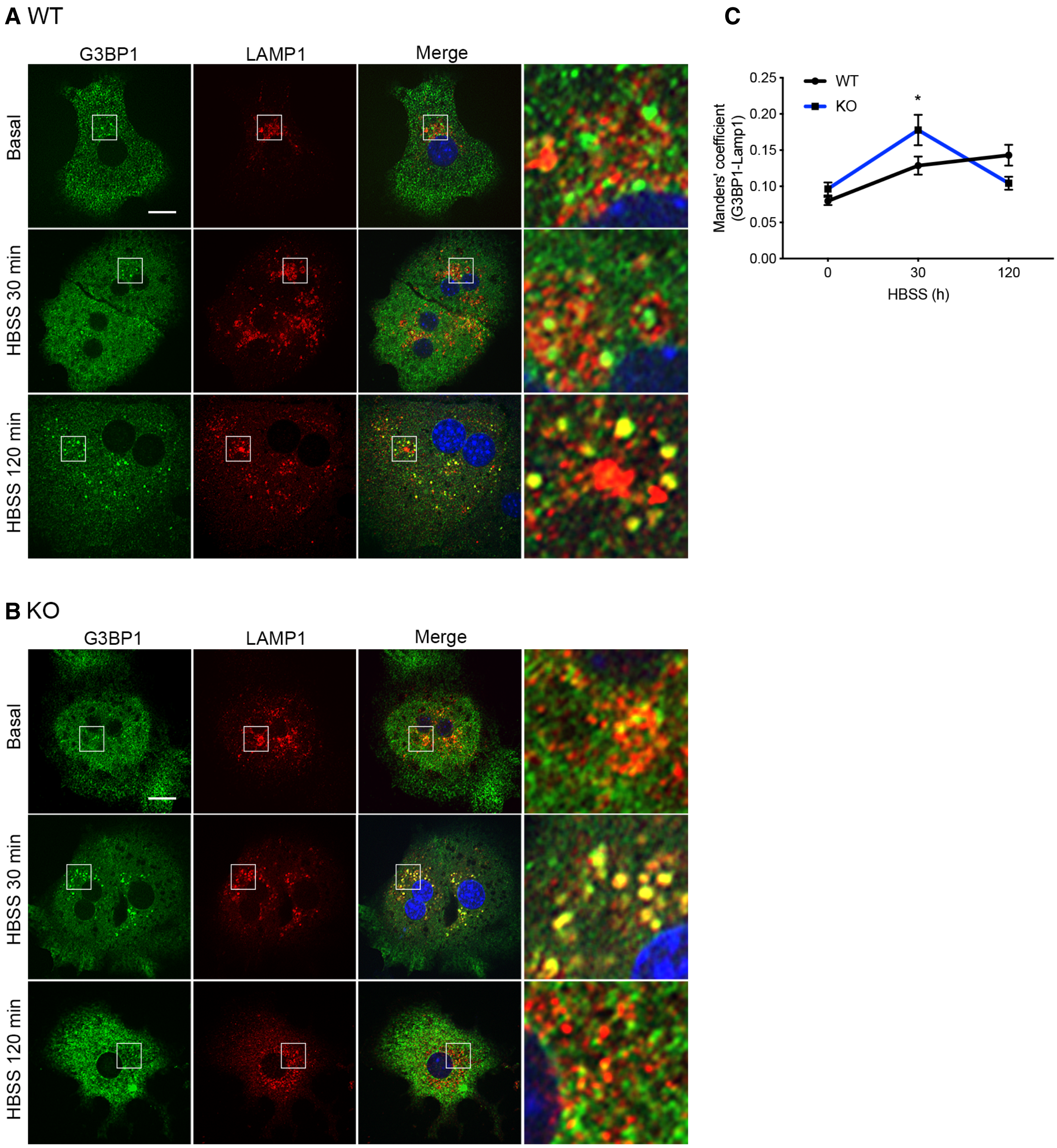

Figure EV3. G3BP1 granules colocalize with LAMP1.

A, B Confocal images of primary hepatocytes isolated from Li-*Cluh*^{WT} (A) and Li-*Cluh*^{KO} (B) mice and stained with anti-G3BP1 and anti-LAMP1 antibodies. 7× magnified areas are shown on the right side. Scale bar, 10 μm.

C Manders' colocalization coefficient between G3BP1 and LAMP1 signals of experiments shown in (A, B) (n = 35 cells with granules from 3 mice per genotype).

Data information: In (C), data show the mean ± SEM. (C) *$P \le 0.05$ (Student's t-test); ***$P \le 0.001$ for time; and **$P \le 0.01$ for interaction time–genotype (two-way ANOVA).

© 2020 The Authors

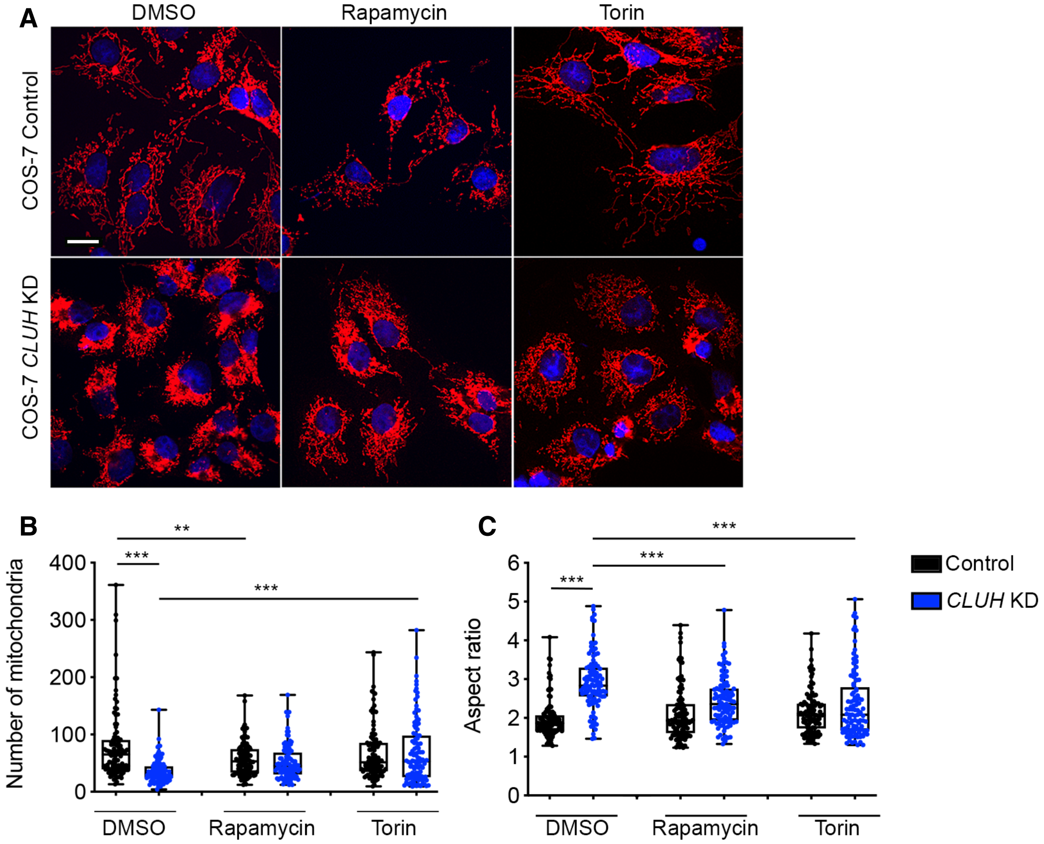

Figure EV4. Mitochondrial clustering in the absence of CLUH is recovered by mTORC1 inhibition.

A Confocal images of COS-7 cells downregulated for CLUH and treated with rapamycin or torin and stained with anti-TOM20 antibody. Scale bar, 10 μm.

B, C Quantification of morphological parameters of experiments shown in (A) (n > 100 cells analyzed from 3 independent experiments).

Data information: In (B, C), data are presented as boxplots showing the median, the first quartile, and the third quartile. Error bars show minimum and maximum values. (B, C) $^{**}P \leq 0.01$; $^{***}P \leq 0.001$ (one-way ANOVA, Tukey's multiple comparison test).

 © 2020 The Authors

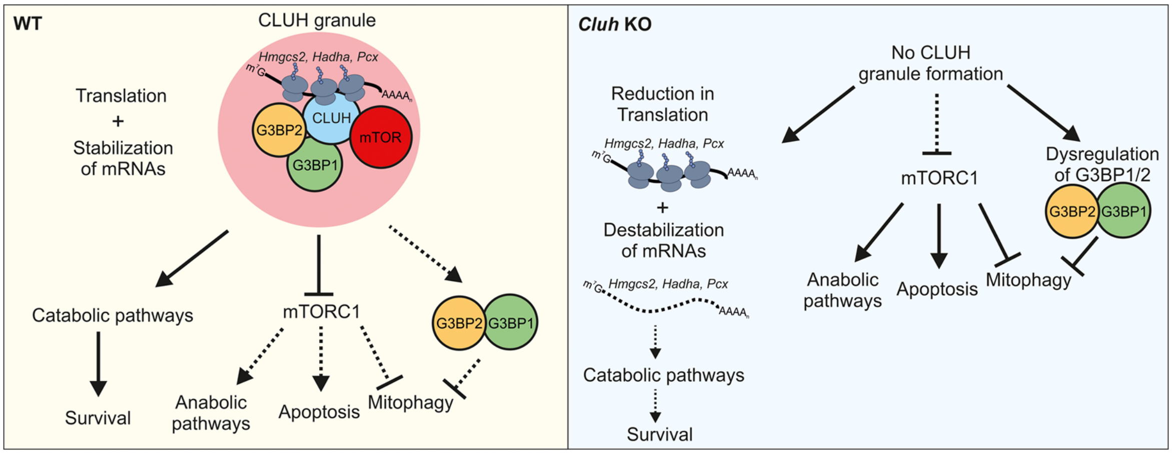

Figure EV5. Model of CLUH function in the liver.
CLUH forms granules containing target mRNAs and regulates their translation. In addition, CLUH granules recruit G3BP1, G3BP2, and mTOR, thereby enhancing mitophagy and inhibiting mitochondrial anabolic pathways. Together, these roles of CLUH are crucial in the liver to survive starvation. In the absence of CLUH, CLUH granules do not form resulting in reduced translation of *Hmgcs2*, *Hadha,* and *Pcx* mRNAs, in failure to suppress mTORC1-dependent anabolic pathways, and in a block in mitophagy.

© 2020 The Authors
