# Supplementary figures and images for: CLUH granules coordinate translation of mitochondrial proteins with mTORC1 signaling and mitophagy
Source: EMBO J. 2020 Mar 9;39(9):e102731. doi: 10.15252/embj.2019102731 (PMC7196838; doi:10.15252/embj.2019102731)

Figure 4 A

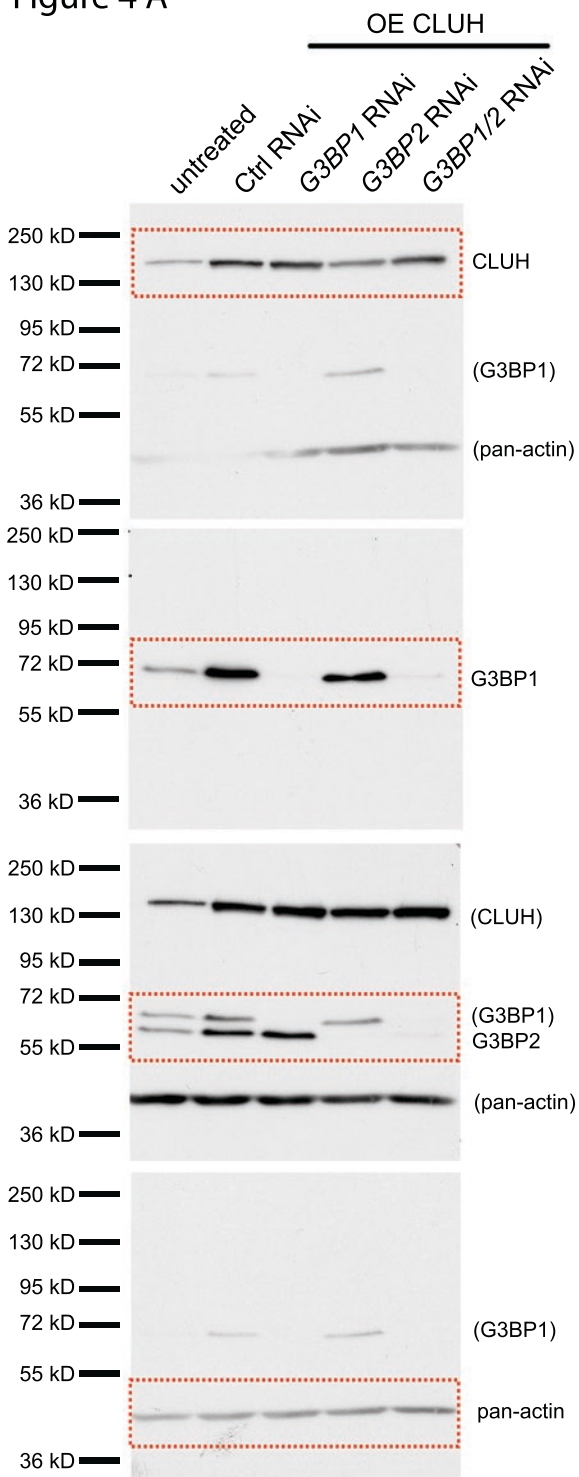

Figure 4 H

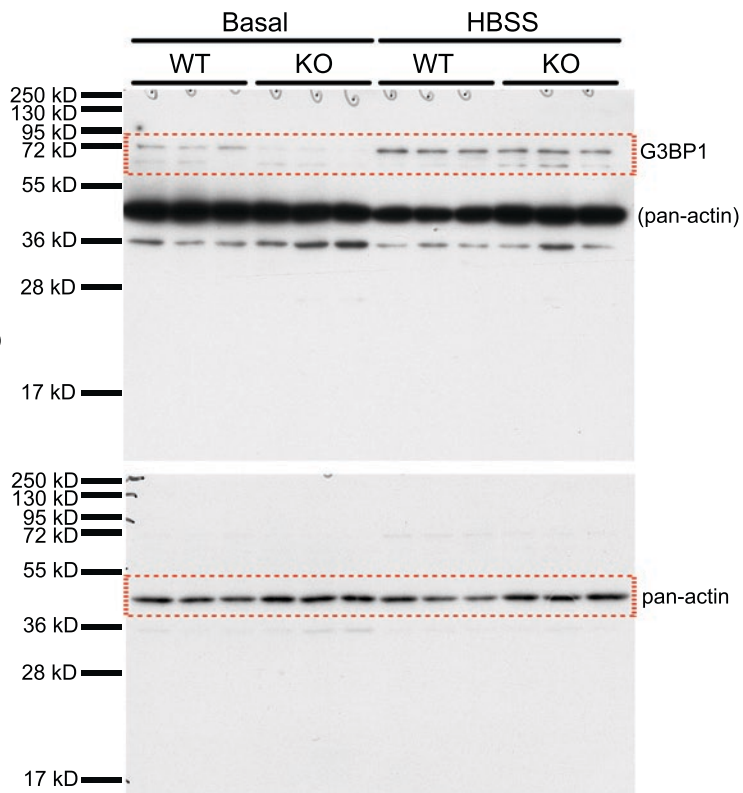

Supplement: Supplementary file 7 — Source Data for Figure 4 [file EMBJ-39-e102731-s006.pdf]

Figure 6A

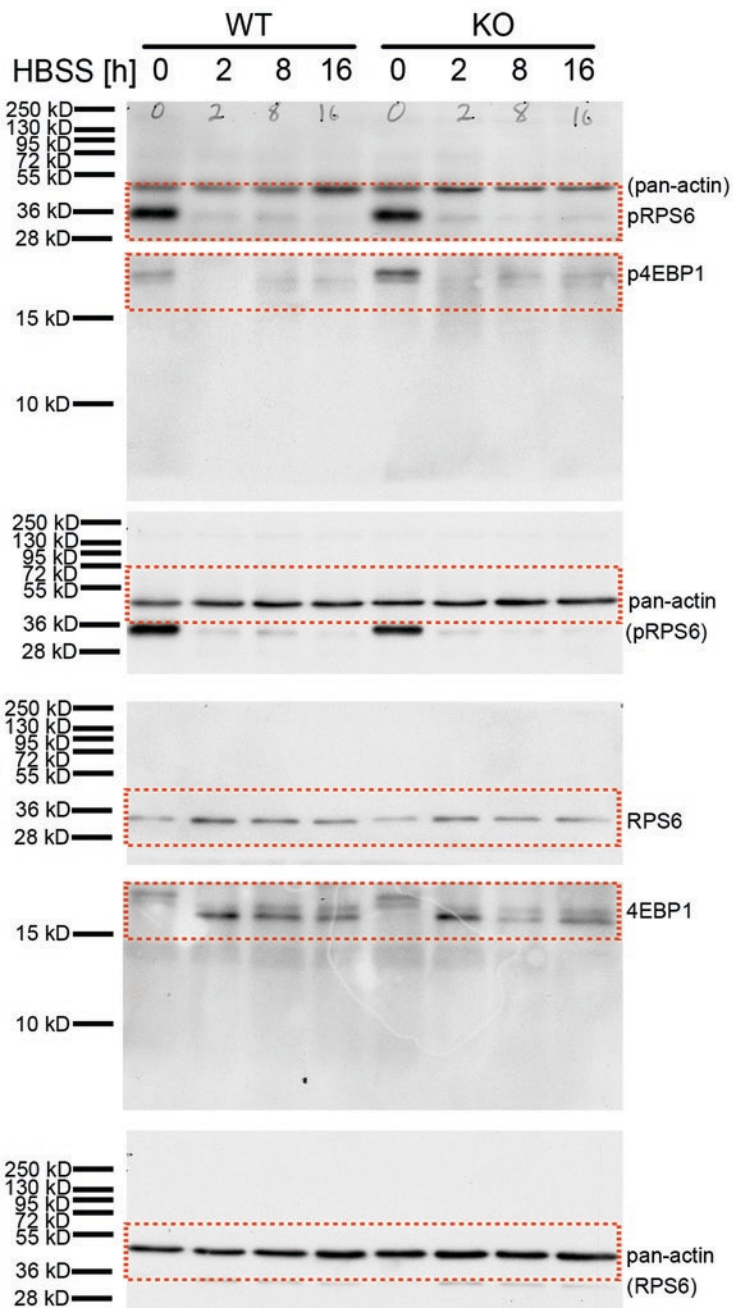

Figure 6D

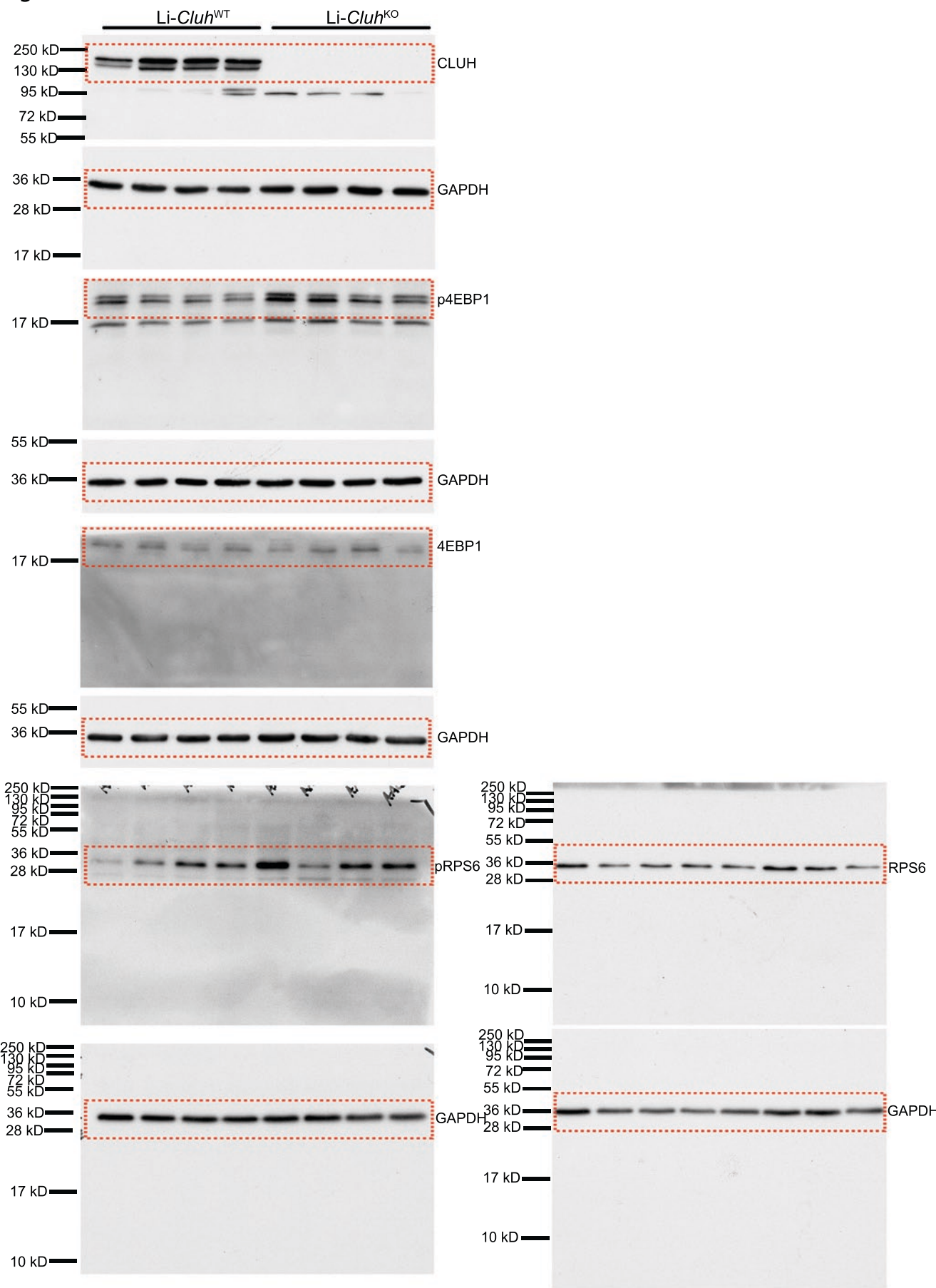

Supplement: Supplementary file 8 — Source Data for Figure 6 [file EMBJ-39-e102731-s007.pdf]

Figure 7F

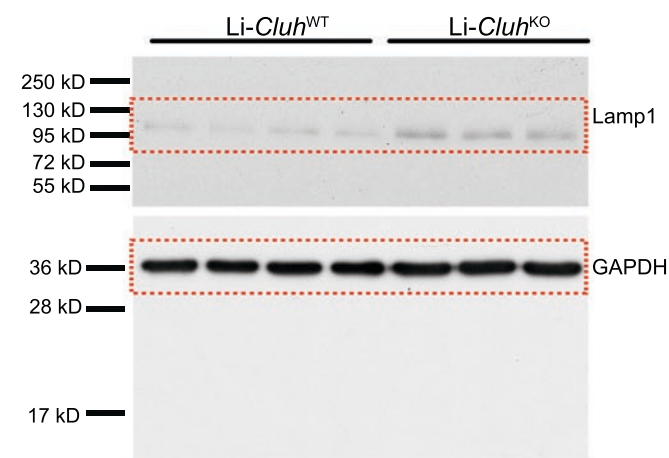

Figure 7H

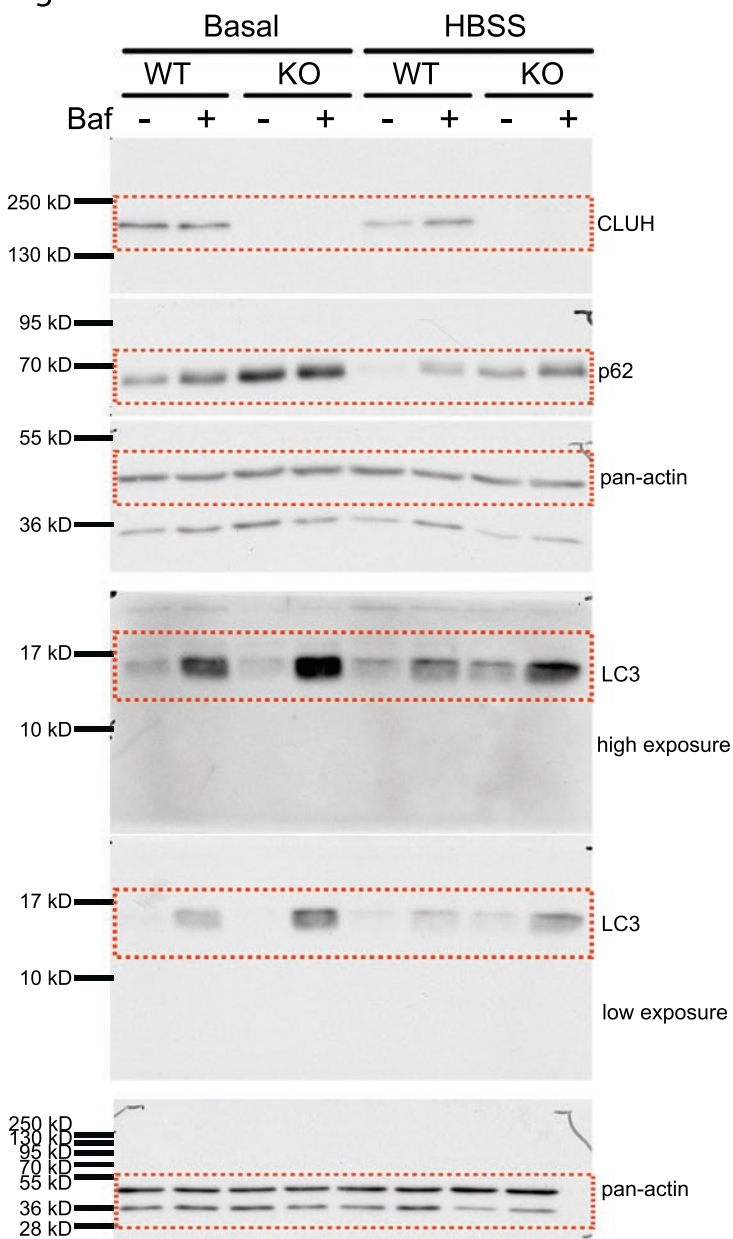

Supplement: Supplementary file 9 — Source Data for Figure 7 [file EMBJ-39-e102731-s008.pdf]

Figure 8A

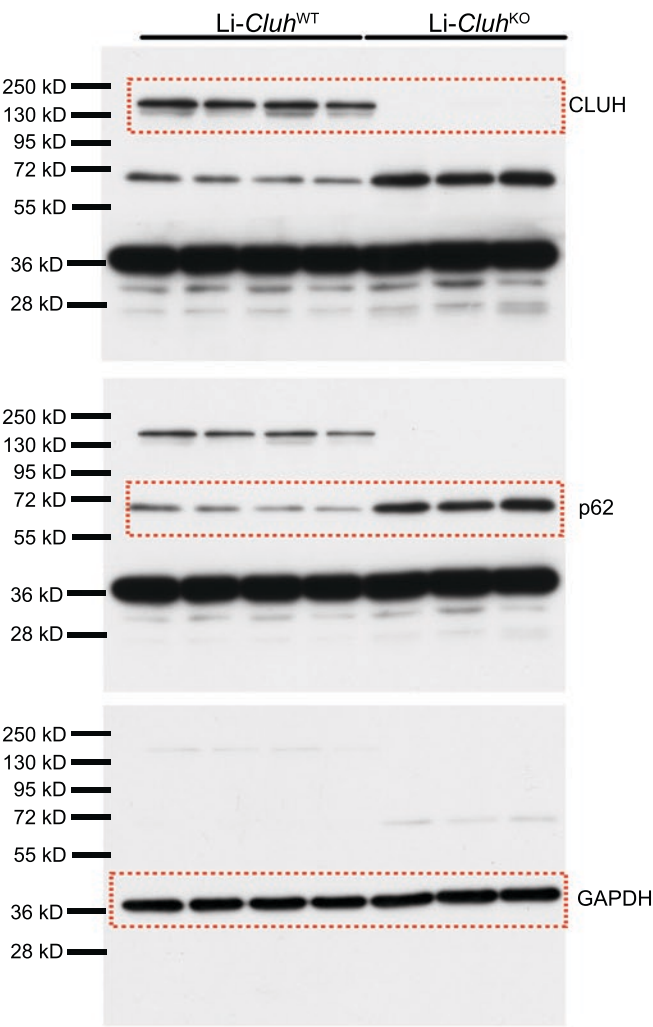

Figure 8C

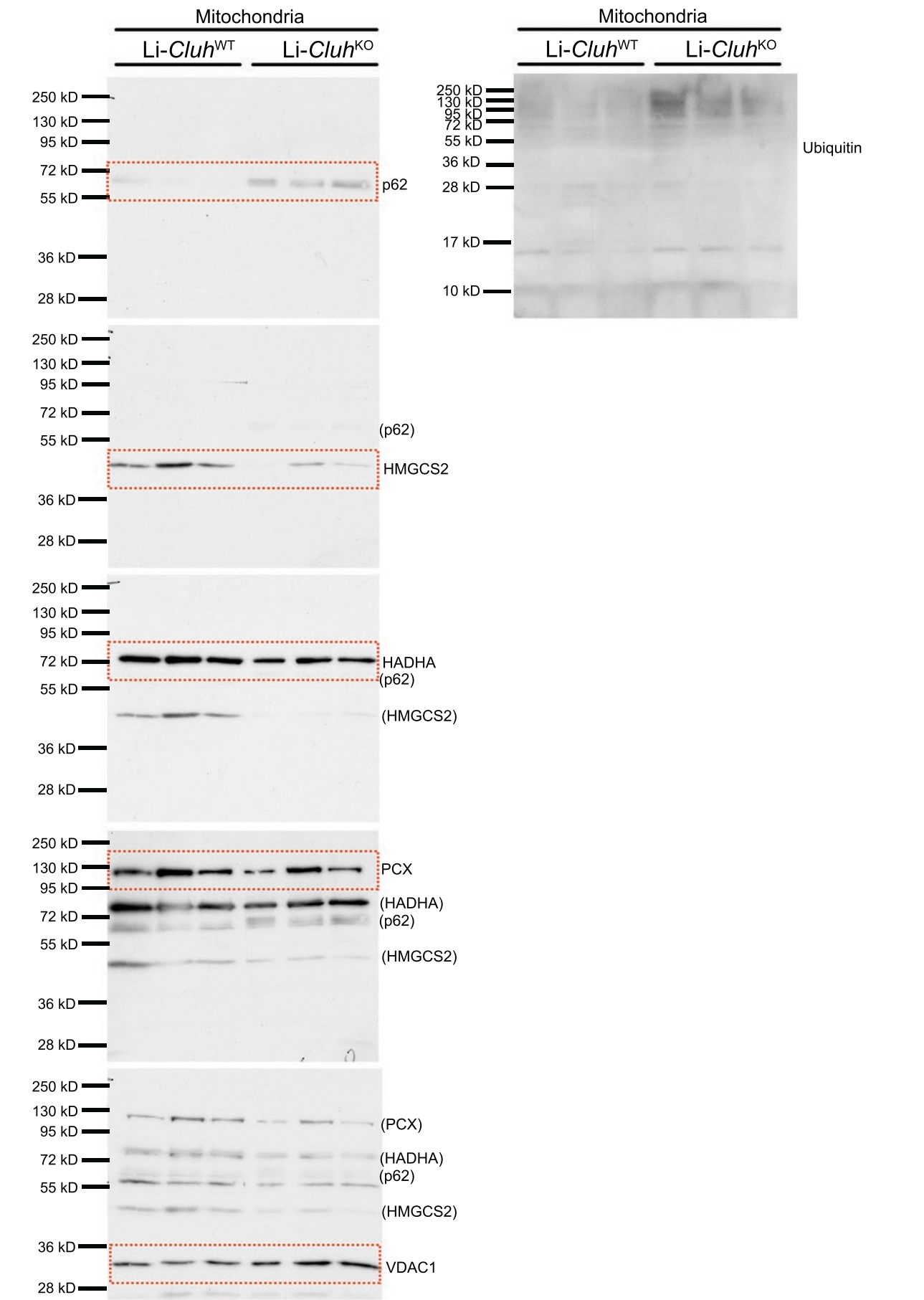

Supplement: Supplementary file 10 — Source Data for Figure 8 [file EMBJ-39-e102731-s009.pdf]

Figure 9F

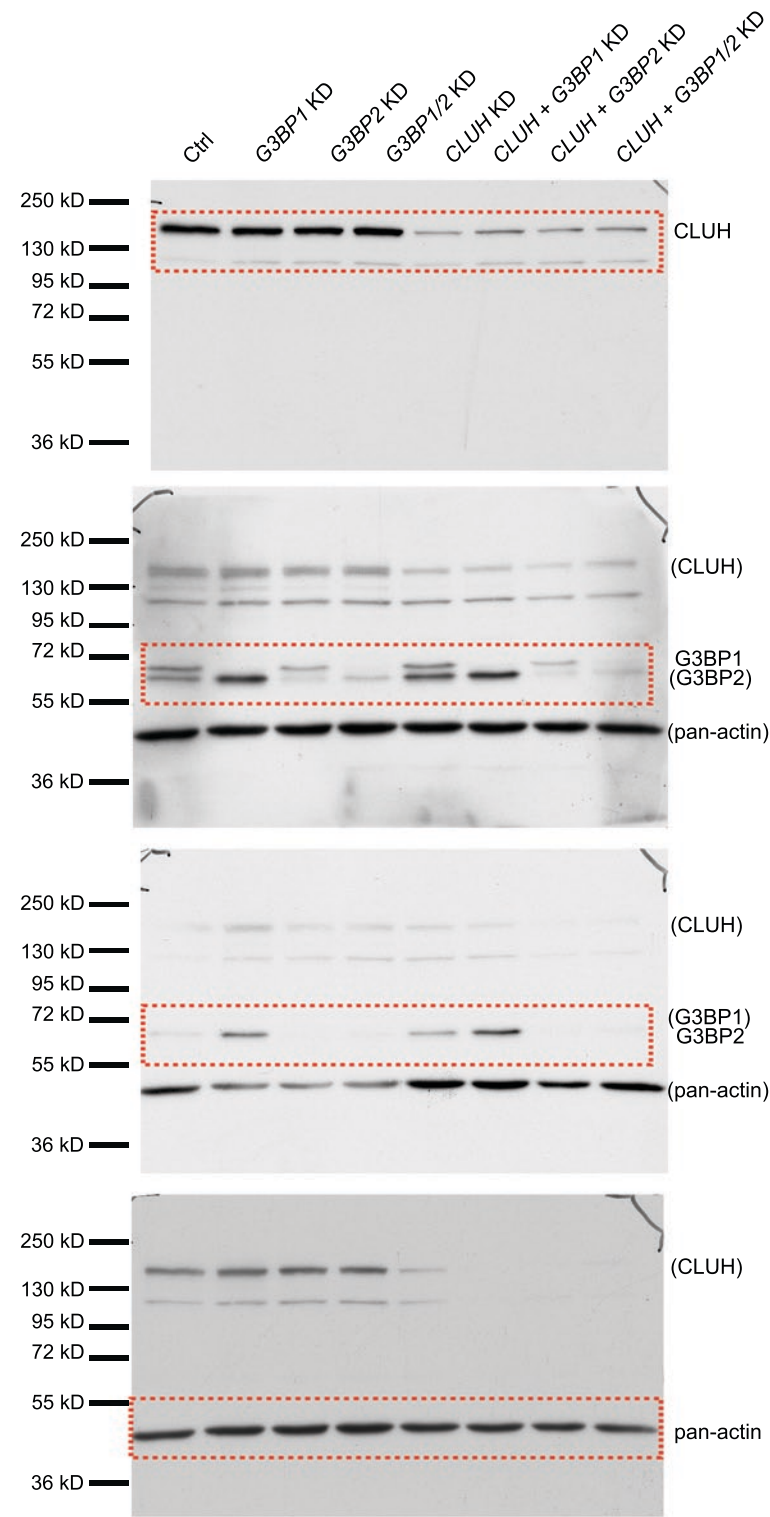

Supplement: Supplementary file 11 — Source Data for Figure 9 [file EMBJ-39-e102731-s010.pdf]
